# P-Glycoprotein Function at the Blood–Brain Barrier: Effects of Age and Gender

**DOI:** 10.1007/s11307-012-0556-0

**Published:** 2012-04-03

**Authors:** Daniëlle M. E. van Assema, Mark Lubberink, Ronald Boellaard, Robert C. Schuit, Albert D. Windhorst, Philip Scheltens, Adriaan A. Lammertsma, Bart N. M. van Berckel

**Affiliations:** 1 Department of Nuclear Medicine & PET Research, VU University Medical Center, PO Box 7057, 1007 MB Amsterdam, the Netherlands; 2 Department of Neurology & Alzheimer Center, VU University Medical Center, PO Box 7057, 1007 MB Amsterdam, the Netherlands; 3 PET Centre, Uppsala University Hospital, 751 85 Uppsala, Sweden

**Keywords:** Aging, Gender, Positron emission tomography, Blood–brain barrier, P-glycoprotein, (*R*)-[^11^C]verapamil

## Abstract

**Purpose:**

P-glycoprotein (Pgp) is an efflux transporter involved in transport of several compounds across the blood–brain barrier (BBB). Loss of Pgp function with increasing age may be involved in the development of age-related disorders, but this may differ between males and females. Pgp function can be quantified *in vivo* using (*R*)-[^11^C]verapamil and positron emission tomography. The purpose of this study was to assess global and regional effects of both age and gender on BBB Pgp function.

**Procedures:**

Thirty-five healthy men and women in three different age groups were included. Sixty minutes dynamic (*R*)-[^11^C]verapamil scans with metabolite-corrected arterial plasma input curves were acquired. Grey matter time–activity curves were fitted to a validated constrained two-tissue compartment plasma input model, providing the volume of distribution (*V*_T_) of (*R*)-[^11^C]verapamil as outcome measure.

**Results:**

Increased *V*_T_ of (*R*)-[^11^C]verapamil with aging was found in several large brain regions in men. Young and elderly women showed comparable *V*_T_ values. Young women had higher *V*_T_ compared with young men.

**Conclusions:**

Decreased BBB Pgp is found with aging; however, effects of age on BBB Pgp function differ between men and women.

## Introduction

The blood–brain barrier (BBB) is composed of a tightly sealed monolayer of brain capillary endothelial cells which, together with astrocytes and pericytes, separates the brain from its external environment. The BBB plays an important role in both protecting the brain and maintaining homeostasis in the central nervous system (CNS) [1, 2]. A key component of the system for regulating the CNS internal milieu is the presence of several transporter systems, which are located at the BBB and are able to transport substances across the BBB [3].

One of the most important transporters at the BBB is the multidrug resistance protein P-glycoprotein (Pgp), encoded by MDR1/ABCB1 and belonging to the family of ATP-binding cassette transporters. Pgp is located throughout the human body in organs or tissues with an excretory and/or barrier function, such as liver, kidney, testes and the BBB [4]. At the BBB, Pgp is highly expressed at the vessel walls of the brain capillaries, where it functions as an efflux pump. Pgp has the remarkable capacity to extrude a large range of structurally and functionally unrelated compounds from the brain. Together with its high expression, this is the main reason that Pgp is considered to be of great importance for protecting the brain from accumulation of potentially toxic substances [5, 6].

Age is a risk factor for many neurodegenerative disorders, such as Alzheimer’s disease (AD) and Parkinsons’s disease (PD) [7, 8]. Progressive dysfunction of Pgp at the BBB with increasing age could be a contributing factor in the increasing risk of developing neurodegenerative disorders with advancing age. For neurodegenerative disorders such as AD, female gender is another risk factor. Several studies have described a higher incidence of AD in women, which might partly be explained by survival differences favouring women, although hormonal differences could also play a role [9–11]. Pgp expression is known to differ between men and women; for example, hepatic Pgp expression is 2.4-fold lower in females, although there are large interindividual differences in Pgp levels [12]. However, little is known about gender differences in Pgp function at the BBB, which again may be under gender specific hormonal influences [13].

Positron emission tomography (PET) with the radiolabelled Pgp substrate (*R*)-[^11^C]verapamil is a validated technique to measure BBB Pgp function *in vivo* [14, 15]. It has been shown that the volume of distribution (*V*_T_) of (*R*)-[^11^C]verapamil inversely reflects cerebral Pgp function [16]. Previous data suggest decreased cerebral Pgp function in healthy aging [17–19] and AD [20], whilst conflicting results have been obtained in PD [21, 22]. The purpose of the present study was to further investigate global and regional effects of age and gender on BBB Pgp function in a large group of healthy controls, as measured using (*R*)-[^11^C]verapamil and PET.

## Materials and Methods

### Participants

Thirty-five healthy male and female subjects in three different age groups (young subjects between 20 and 30 years, middle aged subjects between 40 and 50 years and elderly subjects between 55 and 70 years of age) were recruited through advertisements in newspapers and by means of flyers. Of the 35 subjects, ten males (five young and five elderly) had also been included in a previously published pilot study of Toornvliet et al. assessing BBB Pgp function [17]. Subjects underwent a standardized clinical assessment, including medical history, family history, use of medication and drugs of abuse, and physical and neurological examinations. Subjects had to fulfil research criteria of having never been mentally ill. All subjects had normal scores on screening laboratory tests and urine screening for use of drugs of abuse was negative. All subjects had a normal magnetic resonance imaging (MRI) scan, as evaluated by a neuroradiologist. Mini-Mental State Examination (MMSE) scores were within the normal range (MMSE > 26) [23]. Medication at the time of scanning was not allowed, except for medication known not to interfere with Pgp function [24, 25]. Written informed consent was obtained from all participants after a complete written and verbal description of the study. The study was approved by the Medical Ethics Review Committee of the VU University Medical Center.

### MRI

All subjects underwent structural MRI scans using a 1.0-T Magnetom Impact scanner (Siemens Medical Solutions, Erlangen, Germany) or a 1.5-T Sonata scanner (Siemens Medical Solutions, Erlangen, Germany). Scan protocols on both scanners included an identical coronal T1-weighted 3-D magnetization prepared rapid acquisition gradient echo. Voxel size of the images was 0.98 × 0.98 × 1.49 mm. These MRI scans were used for co-registration and region-of-interest (ROI) definition.

### PET

PET scans were acquired using an ECAT EXACT HR+ scanner (Siemens/CTI, Knoxville, TN, USA) [26]. All subjects received an indwelling radial artery cannula for arterial sampling and a venous cannula for tracer injection. First, a 10-min transmission scan in 2D acquisition mode was performed using three retractable rotating line sources. This scan was used to correct the subsequent emission scan for photon attenuation. Next, a 3D dynamic emission scan was started simultaneously with the injection of 369 ± 19 MBq (*R*)-[^11^C]verapamil, performed using an infusion pump (Med-Rad, Beek, the Netherlands). This emission scan consisted of 20 frames with progressive increase in frame duration (1 × 15, 3 × 5, 3 × 10, 2 × 30, 3 × 60, 2 × 150, 2 × 300, 4 × 600 s; total acquisition time 60 min). During the scan, arterial blood was withdrawn continuously using an online sampling device (Veenstra Instruments, Joure, the Netherlands) [27]. At set times, continuous sampling was interrupted and manual samples were taken. Subject motion was restricted by the use of a head immobilisation device, and position of the head was checked visually at regular intervals during scanning (using laser beams) and corrected immediately when necessary. A more detailed description of scanning and sampling procedures has been reported previously [20].

### PET Data Analysis

All PET sinograms were corrected for dead time, tissue attenuation, decay, scatter and randoms. PET scans were reconstructed using a standard filtered back projection (FBP) algorithm and a Hanning filter with a cutoff at 0.5 times the Nyquist frequency. A zoom factor of 2 and a matrix size of 256 × 256 × 63 were used, resulting in a voxel size of 1.2 × 1.2 × 2.4 mm and a spatial resolution of approximately 6.5 mm full width at half maximum at the centre of the field of view. Images were also reconstructed using a partial volume corrected ordered subset expectation maximization (PVC OSEM) reconstruction algorithm, a previously described and validated method that results in improved image resolution, thereby reducing partial volume effects (PVE) [28–30]. Co-registration of structural T1 MR images to corresponding PET images (using summed FBP or PVC OSEM reconstructed images of frames 3–12) and segmentation of the co-registered MRI into grey matter, white matter and cerebrospinal fluid was performed using statistical parametrical mapping (SPM; version SPM2, www.fil.ion.ucl.ac.uk/spm, Institute of Neurology, London, UK). ROIs were defined on the basis of the segmented MRI and a probabilistic template as implemented in PVElab [31]. Frontal, parietal, temporal, occipital, posterior and anterior cingulate, medial temporal and cerebellar ROIs were used for further analysis. In addition, a global cortical region was defined consisting of the volume weighted average of frontal, parietal, temporal and occipital cortices and posterior and anterior cingulate regions. ROIs were mapped onto dynamic PET images, generating regional time–activity curves.

The original on-line blood curve was calibrated using whole blood radioactivity concentrations derived from the seven manual samples. The calibrated whole blood curve was multiplied with a single-exponential fit to the plasma-to-whole blood ratios of these samples, thereby generating a total plasma curve. Finally, the metabolite corrected plasma input function was obtained by multiplying this total plasma curve with a sigmoid fit to one minus the polar metabolite fraction [16, 32]. This method of obtaining the plasma input function for (*R*)-[^11^C]verapamil, corrected for polar metabolites, has been described in more detail previously [17].

Kinetic analyses were performed using software developed within Matlab 7.04 (The Mathworks, Natick, MA, USA). (*R*)-[^11^C]verapamil data were analysed using a standard two tissue compartment model with the regional *K*_1_/*k*_2_ ratio fixed to the value obtained for the whole brain grey matter ROI. The volume of distribution (*V*_T_) was used as outcome measure. Again, data analysis and kinetic modelling procedures have been described in detail elsewhere [20].

### Statistical Analysis

Statistical analyses were performed using SPSS 15.0 (SPSS Institute, Chicago, IL, USA). Values are presented as mean ± standard deviation. Potential differences in injected tracer dose, specific activity and tracer metabolism (parent and polar metabolite fraction) were verified using Students *t* tests. Comparison of global and regional differences in *V*_T_ between age and gender groups was assessed using both Students *t* tests and analysis of variance (ANOVA) with post hoc least significance difference (LSD) testing. Linear regression analyses were performed using *V*_T_ as dependent variable and age (continuous) as independent variable. The threshold for significance was set at *p* < 0.05.

## Results

Thirty-five subjects were included in the study, 16 women and 19 men, divided into three different age groups: young (age 24 ± 2, range 21–27 years), middle aged (age 46 ± 3, range 42–50 years) and elderly (age 63 ± 4, range 57–69 years) (Table 1). Four of the female subjects (two young, two middle aged) were on oral contraceptives, which were all stopped in the week of PET scanning. The middle-aged women included in analyses were aged 43, 43, 44 and 48, respectively, and were not in the menopause or had reached postmenopausal status yet. There was no use of hormonal replacement therapy in female subjects and no use of other medication in both male and female participants. There were no differences in injected tracer dose and specific activity between the age groups, gender groups or gender groups within the age groups. One subject (female, middle aged group, 43 years) was excluded from further analysis because of technical problems during arterial sampling.

**Table 1:** Characteristics of age groups

	*n*	Female (%)	Age (years)	ID (MBq)	SA (GBq **·** μmol^−1^)
Young	9	44	24 ± 2	369 ± 14	51 ± 16
Middle	10	50	46 ± 3	368 ± 15	51 ± 22
Old	16	44	63 ± 4	371 ± 35	39 ± 13

*n* number of subjects, *ID* injected dose of (*R*)-[^11^C]verapamil, *SA* specific activity of the injected (*R*)-[^11^C]verapamil

Areas under the curve (AUCs) for both parent and polar metabolite fractions of (*R*)-[^11^C]verapamil were calculated. Parent fraction AUCs were 38.0 ± 4.1, 35.9 ± 5.1 and 39.5 ± 4.6 min for young, middle aged and elderly subjects, respectively. Polar metabolite fraction AUCs were 10.0 ± 3.2, 8.9 ± 2.5 and 9.3 ± 2.2 min for young, middle aged and elderly subjects, respectively. In summary, there were no significant differences in tracer metabolism between the age groups. Similarly, within each age group, there were no significant differences in metabolism between males and females.

For FBP reconstructed data, significant (*p* < 0.05) increases in (*R*)-[^11^C]verapamil *V*_T_ were found in the elderly group compared with the young group for frontal, temporal, posterior and anterior cingulate, medial temporal lobe and cerebellar regions (Table 2). For PVC OSEM reconstructed data, similar results were obtained, except that in this case, also a significant increase in *V*_T_ of the global cortical region was seen (Table 3). No significant differences were seen between young and middle aged groups and between middle aged and elderly groups. As effects of PVE correction on (*R*)-[^11^C]verapamil data were minimal (Tables 2 and 3), all further analyses were performed for FBP reconstructed data.

**Table 2:** Volume of distribution of (*R*)-[^11^C]verapamil in several brain regions for different age groups in case of FBP reconstructed data

	Young	Middle	Old
Global	0.71 ± 0.2	0.75 ± 0.1	0.84 ± 0.2
Frontal	0.71 ± 0.2	0.74 ± 0.1	0.85 ± 0.2*
Parietal	0.70 ± 0.2	0.75 ± 0.1	0.83 ± 0.2
Temporal	0.71 ± 0.2	0.76 ± 0.1	0.86 ± 0.2*
Occipital	0.73 ± 0.2	0.76 ± 0.1	0.85 ± 0.2
Posterior cingulate	0.65 ± 0.1	0.74 ± 0.1	0.82 ± 0.2*
Anterior cingulate	0.66 ± 0.2	0.72 ± 0.1	0.80 ± 0.1*
Medial temporal	0.77 ± 0.2	0.88 ± 0.2	1.07 ± 0.3*
Cerebellum	0.67 ± 0.2	0.74 ± 0.1	0.88 ± 0.2*

*p < 0.05 for young *versus* elderly aged group

**Table 3:** Volume of distribution of (*R*)-[^11^C]verapamil in several brain regions for different age groups in case of PVC OSEM reconstructed data

	Young	Middle	Old
Global	0.68 ± 0.2	0.73 ± 0.1	0.82 ± 0.2*
Frontal	0.68 ± 0.2	0.72 ± 0.1	0.84 ± 0.2*
Parietal	0.67 ± 0.2	0.72 ± 0.1	0.81 ± 0.2
Temporal	0.68 ± 0.2	0.75 ± 0.1	0.83 ± 0.2*
Occipital	0.69 ± 0.2	0.73 ± 0.1	0.81 ± 0.1
Posterior cingulate	0.64 ± 0.1	0.73 ± 0.1	0.82 ± 0.2*
Anterior cingulate	0.65 ± 0.2	0.72 ± 0.1	0.79 ± 0.1*
Medial temporal	0.76 ± 0.1	0.91 ± 0.2	1.09 ± 0.4*
Cerebellum	0.65 ± 0.2	0.72 ± 0.1	0.80 ± 0.1*

*p < 0.05 for young *versus* elderly aged group

**Fig. 1:**
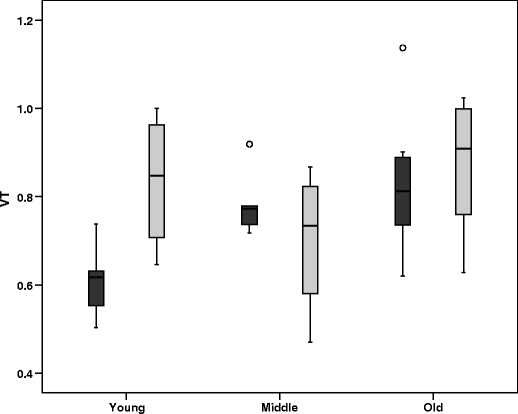
Boxplot of volume of distribution (*V*_T_) of (*R*)-[^11^C]verapamil for the global cortical brain region for the young, middle aged and elderly groups. Men are represented by *dark grey bars*, women by *light grey bars*. *Open circles* outliers, VT = *V*_T._

Within each age group, the effect of gender was assessed. Results for the global cortical region are shown in Fig. 1, illustrating a significantly higher mean *V*_T_ in young women than in young men, but no differences in the other age groups. This also implies that age effects are different for males and females. Therefore, ANOVA was performed to assess age effects for each gender separately. For men, a main effect of age was found for all regions, including the global cortical region. Post hoc LSD analyses revealed significant differences in *V*_T_ between young and middle aged groups for all regions, except frontal and cerebellar ROI. In addition, significant differences between young and elderly groups were observed for all brain regions. In contrast, in women, no main effect of age was seen in any of the regions. Finally, for men and women separately, the relationship between (*R*)-[^11^C]verapamil *V*_T_ and age was assessed using linear regression analysis. Regression coefficients and *p* values are given in Table 4. Again, there was a main effect of age in men for all regions, but none in women.

**Table 4:** Regional regression coefficients (and corresponding *p* values) for linear regression of the volume of distribution of (*R*)-[^11^C]verapamil against age in men and women separately

	Men	Women
Global	0.64 (0.003)	0.12 (0.672)
Frontal	0.63 (0.004)	0.10 (0.732)
Parietal	0.63 (0.004)	0.10 (0.734)
Temporal	0.65 (0.003)	0.24 (0.465)
Occipital	0.62 (0.005)	0.04 (0.891)
Posterior cingulate	0.58 (0.009)	0.21 (0.443)
Anterior cingulate	0.66 (0.002)	0.14 (0.620)
Medial temporal	0.68 (0.001)	0.42 (0.123)
Cerebellum	0.60 (0.006)	0.30 (0.273)

## Discussion

This is the first study to assess *in vivo* effects of gender and aging on human BBB Pgp function in a large sample size with PET and (*R*)-[^11^C]verapamil. This study shows an 18 to 38 % increase in the volume of distribution of (*R*)-[^11^C]verapamil with normal aging in several brain regions, such as frontal, temporal, medial temporal and anterior and posterior cingulate regions. These data are consistent with an age-related decline in BBB Pgp function and are in line with previous studies assessing BBB Pgp function *in vivo* during healthy aging [17–19]. Importantly, the present results indicate that the effects of age on BBB Pgp function are driven by men. In women, no main effect of age on Pgp function was found.

Using PET and [^11^C]verapamil, three previous studies have assessed BBB Pgp function in healthy aging. In a pilot study in ten subjects, five young and five elderly males, Toornvliet and colleagues observed an ~18 % decrease in Pgp function in the elderly for a whole brain grey matter region of interest [17]. Bartels and colleagues were the first to study regional differences in Pgp function with aging and found clusters with higher uptake in white matter regions in ten elderly compared with seven younger subjects (14 males) using a voxel-based approach [18]. Bauer and colleagues extended these findings in thirteen subjects (11 males), in which six older subjects had increased tracer uptake in several brain regions, although some of these findings disappeared after correcting for PVE [19]. These previous studies used relatively small subject groups and did not take into account effects of gender on Pgp function. Furthermore, not all of these studies used the pure enantiomer (*R*)-[^11^C]verapamil, which is the preferred tracer for quantifying Pgp function [16, 33, 34]. In addition, some of these studies have used single-tissue compartment models [17, 19] whilst recent studies have shown that two components can be identified in (*R*)-[^11^C]verapamil data [20, 35, 36]. As such, a two-tissue compartment model provides better fits to (*R*)-[^11^C]verapamil data [16, 37].

This difference in age effects on BBB Pgp function seems to be mainly due to the relatively high distribution volumes in young female subjects. The reason for this remains unknown, but it could be due to interindividual differences in Pgp function itself, or interindividual differences in (fluctuating) hormone levels in women that, in turn, could affect Pgp function. It is known that (both endogenous and synthetic) progesterone/progestins and estrogens can have an effect on Pgp function [13]. In an attempt to limit these individual differences in (fluctuating) hormone levels, all female subjects in both young and middle aged groups were scanned at comparable time points in their menstrual cycle (during menstruation). Of course, this does not guarantee comparable hormone levels or comparable effects of hormone levels on Pgp function. In the present study, these hormone levels were not measured. Further studies are needed to investigate the relationship between (*R*)-[^11^C]verapamil measurements and actual hormone levels. Interestingly, a preclinical study has found differences in brain uptake of verapamil comparing female mice to male mice, which suggested a modest increase in Pgp expression and/or function in female animals [38]. This is in contrast to the findings of the present clinical study in humans, in which a higher *V*_T_ was found in young women than in young men, suggesting reduced Pgp function in young women. Further studies are needed to assess whether this discrepancy is due to differences in hormonal status or species differences in transporter expression and/or activity at the BBB. Using PET and (*R*)-[^11^C]verapamil, it is not possible to differentiate between decreased Pgp function due to a decrease in BBB Pgp expression or due to decreased functionality of the transporters with intact Pgp expression.

Decreased Pgp function with increasing age could account for increased drug toxicity and increased CNS side effects of drugs that are able to pass the BBB in the elderly. Additionally, older people more often suffer from health problems, and it is not uncommon that this results in polypharmacy which often includes the use of Pgp inhibiting drugs, which may lead to further impairment of the protective function of Pgp. Furthermore, a decrease in Pgp function with increasing age could make the elderly more vulnerable to both exogenous as well as endogenous neurotoxins that are transported by Pgp (such as amyloid-beta, the protein that accumulates in the brain in AD), and this may contribute to the increasing risk of neurodegenerative diseases with age. Results of this study suggest decreased Pgp function in young women compared with young men, which could implicate that women are exposed to higher concentrations of neurotoxins earlier in life and therefore during a longer time period, which in turn could possibly account for the increased risk of AD in women. Recently, using PET and (*R*)-[^11^C]verapamil, it has been shown that AD patients have diminished BBB Pgp function compared with healthy elderly aged subjects, further supporting a possible role of Pgp in the aetiology of AD [20].

Data in this study were assessed with and without a PVE correction method, showing comparable results in distribution volumes. It should be noted that, although the age range varied from 21 to 69 years, there was no significant brain atrophy present on MRI scans, and as (*R*)-[^11^C]verapamil is a tracer which has low uptake throughout the brain and therefore shows little contrast, no major effects from PVE correction methods should be expected.

Strengths of the present study are the relatively large sample size of the three different age groups and the wide age range of subjects included, in addition to the quantitative nature of the study. Nevertheless, a limitation of the study is that the different age groups still become relatively small when separated according to gender. In addition, despite extensive screening, inclusion of subjects with preclinical neurodegenerative disorders, which by themselves are associated with decreased Pgp function [20], cannot be excluded.

## Conclusions

The volume of distribution of (*R*)-[^11^C]verapamil increases with age in several cortical brain regions, strongly suggesting a progressive decrease in BBB Pgp function with age. However, this effect is only seen in males, suggesting different aging patterns between men and women. This finding highlights the need to include both males and females in aging studies.
